# Ligation of the Maxillary Artery Prior to Caudal Maxillectomy in the Dog—A Description of the Technique, Retrospective Evaluation of Blood Loss, and Cadaveric Evaluation of Maxillary Artery Anatomy

**DOI:** 10.3389/fvets.2020.588945

**Published:** 2020-11-05

**Authors:** Kenneth A. Carroll, Kyle G. Mathews

**Affiliations:** Department of Clinical Sciences, College of Veterinary Medicine, North Carolina State University, Raleigh, NC, United States

**Keywords:** caudal maxillectomy, preligation, hemorrhage, blood loss, maxillary artery ligation

## Abstract

Two different surgical techniques have been described for performing caudal maxillectomies in dogs including the intraoral (IO) and combined dorsolateral and intraoral (DL-IO) approach. Hemorrhage is the most common intraoperative complication reported during these procedures as maxillary arterial ligation is not performed until after all osteotomies and mobilization of tumor-bearing bone. The objectives of this study were to describe a modified approach for caudal maxillectomy in the dog involving preligation of the maxillary artery, to retrospectively evaluate the ability of this modified approach to limit hemorrhage in a cohort of 22 dogs, and to clarify the vascular anatomy of the maxillary artery and its branches in relation to associated nerves. Medical records were retrospectively reviewed for cases that had caudal maxillectomy via a combined approach (with or without preligation of the maxillary artery) from January 1, 2004 to December 31, 2019. Twenty-two cases were identified, six without, and 16 with arterial preligation, respectively. Osteotomies were completed with a high-speed handpiece and rotary bur (*n* = 18), or oscillating bone saw (*n* = 4). All six (100%) dogs in the traditional DL-IO group developed hypotension under general anesthesia. Four (67%) of these required intraoperative blood transfusions, one of which required an additional postoperative blood transfusion. In contrast, only one of 16 (6%) dogs in the modified DL-IO group required an intraoperative blood transfusion, and only three (19%) developed hypotension. Moreover, a significant association was detected between postoperative PCV and the two different surgical approaches (*P* = 0.021). These results demonstrate the effectiveness of preligation of the maxillary artery in preventing hemorrhage in caudal maxillectomies in dogs and this represents an improvement in outcome over previously reported studies. Decreased intraoperative hemorrhage may improve surgical exposure and decrease overall patient morbidity.

## Introduction

The oral cavity is a common location for the development of neoplasia in small animals (1, 2). Caudal maxillectomy is often part of the treatment plan for caudally located oral neoplasms that have not yet crossed the midline of the hard palate (3, 4). It is common for these tumors to be large in size, as it can be difficult for owners to recognize tumor growth in this location (5).

Two different surgical techniques have been described for performing a caudal maxillectomy in dogs including intraoral (IO) and combined dorsolateral and intraoral (DL-IO) approaches. The IO approach was first described in 1985 (6, 7) and is recommended for unilateral benign and malignant tumors located along the alveolar margins of the mid-to-caudal maxilla (8). While this technique is effective for tumors adjacent to the dental arch, visualization becomes difficult for larger tumors in this area, especially for more dorsal or caudally located neoplasms (9). The combined DL-IO approach was described in 2003 as a modification of the existing procedure (9). This technique is recommended for tumors of the mid-to-caudal maxilla that arise or extend dorsolaterally and/or caudally into the inferior orbit, and provides improved exposure and thus increased ability to resect the mass to microscopic disease and potentially achieve clean surgical margins (8, 9).

**Figure 1:**
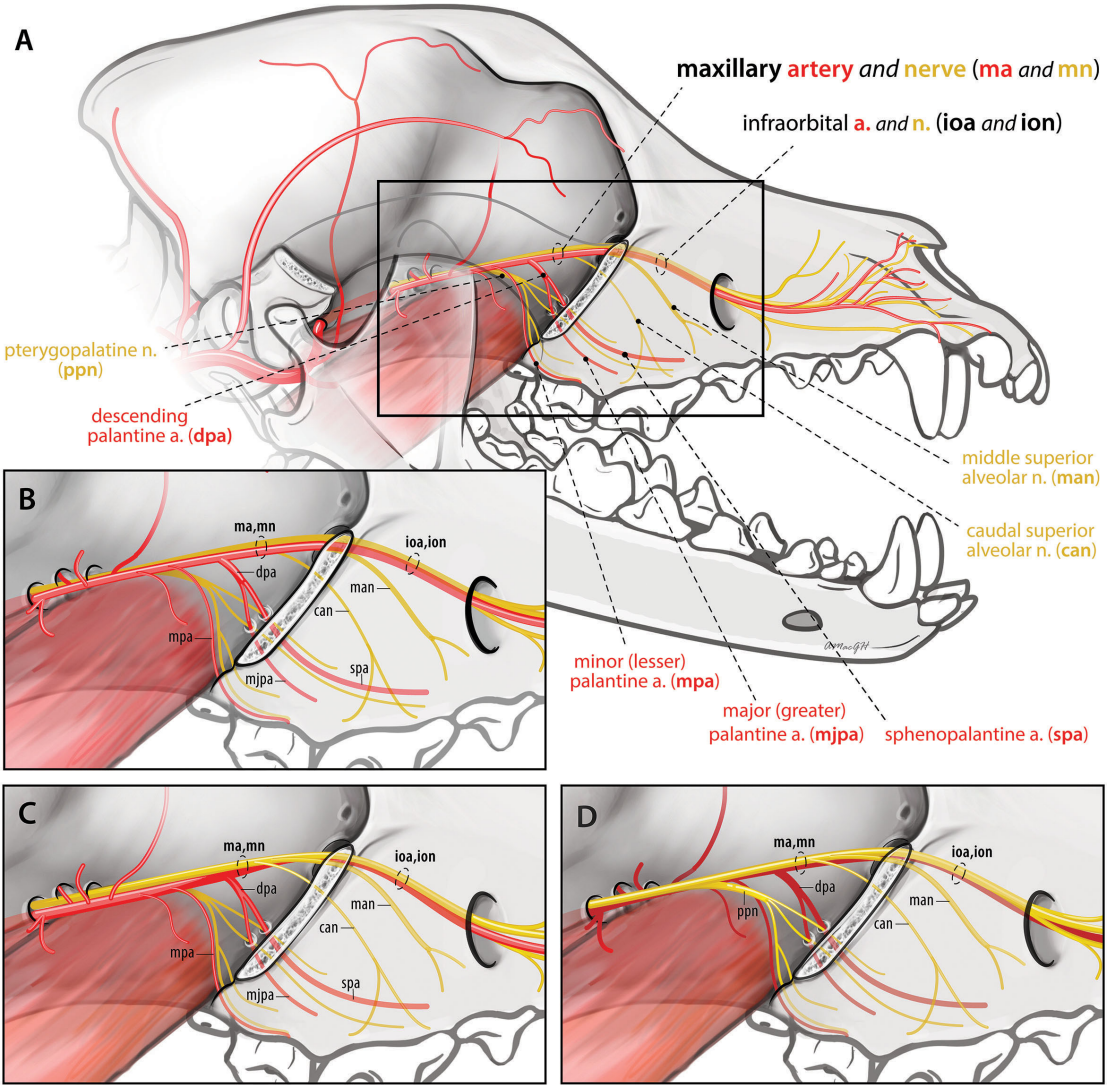
Spatial relationship of the maxillary nerve and artery branches caudal to the infraorbital canal. **(A,B)** (inset) Version 1: Arterial branches remain lateral to nerve branches throughout (*n* = 7). Maxillary artery (ma), minor palatine artery (mpa), descending palatine artery (dpa), infraorbital artery (ioa), major palatine artery (mjpa), sphenopalatine artery (spa), maxillary nerve (mn), caudal superior alveolar nerve (can), middle superior alveolar nerve (man), infraorbital nerve (ion). **(C)** Version 2: The infraorbital artery rostral to the descending palatine artery runs ventral and then medial to the nerve prior to entering the infraorbital canal (*n* = 12). **(D)** Version 3: All arterial branches lay medial to the maxillary, caudal superior alveolar, middle superior alveolar and infraorbital nerves (*n* = 1).

The maxillary nerve is a branch of the trigeminal nerve and as such contains sensory fibers (10). After exiting the rostral alar foramen into the caudal aspect of the orbit, the maxillary nerve gives off the pterygopalatine nerve ventromedially which in turn gives rise to the minor palatine, major palatine and caudal nasal nerves [(10–12); Figure 1]. Both the maxillary and pterygopalatine nerves run rostrally on the dorsolateral surface of the medial pterygoid muscle along the medial aspect of the orbit. Further rostrally, after giving off the caudal superior alveolar and caudal nasal nerve branches ventrally, the maxillary nerve becomes the infraorbital nerve caudal to the maxillary foramen (entrance to the infraorbital canal). This area just caudal to the maxillary foramen is where a maxillary nerve block would occur (10).

Blood supply to the maxilla is provided by four branches of the maxillary artery: the major and minor palatine, sphenopalatine, and infraorbital arteries (11). The maxillary artery is the main continuation of the external carotid artery (11). In the rostral aspect of the orbit, the maxillary artery gives off the minor palatine artery ventrally, and this then divides into the similarly sized infraorbital and descending palatine arteries [(11, 12); Figure 1]. The descending palatine artery subsequently divides into the major palatine and sphenopalatine arteries ventrally, while the infraorbital artery continues rostrally through the maxillary foramen to enter the infraorbital canal.

Because of the maxillary artery's location, it is typically not ligated until after the majority of tumor resection has been completed during caudal maxillectomies (3, 8, 9). Regardless of bone cutting device (e.g., osteotome and mallet, oscillating bone saw, high speed rotary bur, or piezoelectric oral surgery unit), hemorrhage is the most common intraoperative complication reported during caudal maxillectomy (4). Intraoperative hemorrhage requiring transfusion was noted in six of 20 (30%) dogs in one study (9), 44 of 103 (42.7%) dogs in a second study (4), and one of two (50%) dogs in another study (5). Additionally, one dog died of hemorrhage and hypovolemic shock following caudal maxillectomy in another study (13). In each of these studies, an oscillating bone saw was the predominant bone cutting device used.

According to the initial description of the DL-IO approach, if bleeding is encountered, this is to be addressed at the end of the osteotomies, following mobilization of the free segment of bone via ligation or vascular clips (9). These patients will inevitably hemorrhage, so speed is the major factor in preventing excessive blood loss, especially when using an oscillating bone saw (9). While temporary occlusion of the ipsilateral carotid artery can be performed (14), no studies have examined the specific effectiveness of preligation of the maxillary artery in preventing blood loss in caudal maxillectomy patients. Additionally, although the anatomy of the maxillary and infraorbital arteries and nerves has been well-described, the relationship of these structures to each other is only briefly mentioned (10, 12). In one “anatomy of the dog” textbook, the maxillary artery and its branches are drawn lateral to the maxillary and infraorbital nerves (10). An understanding of the spatial relationship of these structures caudal to the infraorbital canal could be important to surgeons performing arterial ligation prior to completion of large caudal maxillectomies and to anyone performing maxillary nerve blocks. The objectives of this study were therefore, (1) to describe a modified DL-IO approach for caudal maxillectomy in dogs involving preligation of the maxillary artery, (2) to retrospectively evaluate the ability of this modified approach to limit hemorrhage, and (3) to clarify the vascular anatomy of the maxillary artery and its branches in relation to associated nerves in this area by evaluating latex injected canine cadavers. We hypothesized that ligation of the maxillary artery at the start of caudal maxillectomy would result in less hemorrhage and subsequent need for blood transfusion.

## Materials and Methods

### Part I: Retrospective Evaluation

#### Retrospective Inclusion Criteria

Medical records of dogs undergoing maxillectomy ± orbitectomy at the North Carolina State University Companion Animal Veterinary Medical Center between January 1, 2004 to December 31, 2019 were reviewed. Intraoral maxillectomies (*n* = 69) were not included in this study. Cases without a complete surgical or anesthetic report were excluded from the study. All caudal maxillectomy cases performed via a combined approach with or without preligation of the maxillary artery (*n* = 22) were evaluated for tumor location, regional lymph node involvement and pulmonary metastases via computed tomography (CT) of the head, aspiration of affected lymph nodes and thoracic radiographs. A minimum of 4 weeks postoperative follow-up was necessary for study inclusion.

#### Data Collection

For each dog included in the study, data was collected from the medical record including signalment, weight (kg), clinical signs, date of surgery, tumor size (cm) as measured by CT, tumor location, extent of surgical resection, type of surgical approach (DL-IO vs. modified DL-IO ± orbitectomy and enucleation), duration of surgery (minutes), preoperative PCV (%), intraoperative PCV (%), postoperative PCV (%), requirement of a blood transfusion (including amount), intraoperative complications, immediate postoperative complications (<48 h postoperatively), short-term complications (48 h to 4 weeks postoperatively), histopathological diagnosis (with margins), and adjunctive therapy. Wound dehiscence was defined as the breakdown or opening of an oral wound or suture line, and oronasal fistula formation was defined as an abnormal opening between the oral and nasal cavity.

#### Surgical Procedure

All procedures were performed by a board-certified surgeon assisted by a surgical resident. Surgical approach (IO vs. DL-IO vs. modified DL-IO) was ultimately chosen at the discretion of the attending surgeon. Likewise, anesthetic protocols were determined by the attending anesthesiologist and included a combination of pre-, intra- and postoperative opioids, as well as regional nerve blocks to provide an appropriate level of analgesia throughout the procedure. The DL-IO surgical technique was performed routinely as per previous recommendations (9). However, the modified DL-IO approach included preligation of the maxillary artery at the start of caudal maxillectomy.

#### Modified DL-IO Approach (Preligation of the Maxillary Artery)

All animals were placed in lateral recumbency with the affected side up and prepared for surgery in a routine manner as outlined by the original DL-IO approach (9). Enucleation, if required was performed first. Following enucleation, the maxillary artery and associated nerve were identified caudal to their entrance into the infraorbital canal (maxillary foramen) within the orbit and were occluded with at least two vascular clips (Weck Hemoclip, Teleflex Medical, Research Triangle Park, NC, USA). The remaining procedure was performed as per the original DL-IO description (9). For dogs that did not require enucleation, the skin incision made for the combined approach was extended caudally over the zygomatic arch. Dissection through the subcutaneous tissues and paired levator nasolabialis muscles provided exposure of the zygomatic arch. Angularis oculi and fascial veins were double ligated and transected as they were encountered. The masseter muscle (ventrally) and periosteum were cleared from the zygomatic arch. Rather than performing a single zygomatic osteotomy as previously described (9), two osteotomies were performed and a section of zygomatic arch was removed just caudal to the tumor. Osteotomies were performed using either a high-speed handpiece and rotary bur (Surgairtome Micro100™ Pneumatic, Conmed, Utica, NY, USA), or an oscillating bone saw, and irrigation was provided with continuous lavage of sterile saline. Regional vasculature was approached and cleaned of surrounding soft tissues, primarily periorbital fat, by retracting the eye dorsocaudally with a small malleable retractor. The maxillary artery and nerve were then exposed in the rostral aspect of the orbit using blunt dissection with cotton-tip applicators and right-angled forceps. Vital structures running into the maxillary foramen had vascular clips placed across them. No attempt was made to separate artery from nerve or to locate/identify ventral branches of the maxillary artery (e.g., minor or descending palatine arteries). The maxillary artery was occluded with at least two vascular clips in the rostral aspect of the orbit. The section of the zygomatic arch was submitted for caudal margin histopathological analysis in all 16 cases.

The remainder of the procedure was performed through a combined approach as previously described (9). In brief, the initial dorsal skin incision was extended rostrally to the desired level, and dissection was continued through the subcutaneous tissue, between the paired levator nasolabialis muscles, and down to bone. Periosteum and associated soft tissues were reflected with a periosteal elevator. A second incision was made in the buccal mucosa immediately dorsal to the gingiva. Dissection was continued until connection was made with the skin incision, thereby creating a bipedicle flap of skin, buccal mucosa and associated soft tissues. Retraction of this flap allowed adequate visualization of the lateral aspect of the maxilla and identification of osteotomy locations. Rostral, dorsal and caudal osteotomies were then performed after all soft tissues had been dissected, and residual bleeding was controlled with digital pressure, electrocautery, suture ligation, or hemoclip application. Once all bleeding was controlled, various IO and extraoral reconstructive techniques were used at the discretion of the attending surgeon to achieve a tension-free closure. Further information regarding closure is available in the study describing the original DL-IO approach (9).

Upon recovery, all dogs were given a non-steroidal anti-inflammatory drug such as carprofen (Rimadyl, Zoetis, Parsippany, NJ, USA; 4.4 mg/kg SQ). Postoperatively, they were maintained on a combination of intravenous fluids with lactated Ringer's solution (Baxter Healthcare, Deerfield, IL, USA; 45–60 mL/kg/day), analgesic agents including fentanyl (Generic, Hospira, Lake Forrest, IL, USA; 2–4 μg/kg/h IV) and ketamine (Ketaset, Zoetis, Parsippany, NJ, USA; 0.2–0.5 mg/kg/h IV), and anti-nausea medications such as ondansetron (Mylan, Canonsburg, PA, USA; 0.5 mg/kg IV q8h). PCV, total protein and blood glucose was checked 2 h postoperatively, and overnight, these dogs were monitored closely for any signs of hypotension, facial edema, epistaxis and/or respiratory compromise. Most animals were transitioned onto a transdermal fentanyl patch (Generic, LTS Lohmann, West Caldwell, NJ, USA; 2 μg/kg/h), as well as oral pain medications including carprofen (2.2 mg/kg PO q12h 7 days) and gabapentin (Generic, Strides Pharma, East Brunswick, NJ, USA; 5–10 mg/kg PO q8–12h 14 days), and discharged from the hospital at a minimum of 2 days postoperatively.

### Part II: Evaluation of Cadaveric Maxillary Artery Anatomy

To better define the spatial relationship of the maxillary artery and its branches to the maxillary nerve and thus to assist surgeons with vascular clip placement, cadaveric evaluation was performed after collection of retrospective data. Twenty medium to large breed formalin fixed latex injected canine cadaver heads were evaluated. Heads had been sagittally sectioned prior to fixation (Sargeants Wholesale Biologicals, CA, USA). Ten left and 10 right sided specimens were dissected to expose the maxillary artery and nerve branches along the medial wall of the orbit. The globe, zygomatic arch, lateral wall of the infraorbital canal and orbital soft tissues were removed to improve exposure. The maxillary artery, minor (lesser) palatine artery, major (greater) palatine artery, sphenopalatine artery and infraorbital arteries caudal to the maxillary foramen and within the infraorbital canal were identified. The relationship of these vessels to the maxillary nerve branches was recorded, drawn and photographed (Figure 1).

#### Statistical Analyses

Clinical data was summarized using descriptive statistics (median with range; mean ± SD). Duration of surgery (minutes), preoperative PCV (%), intraoperative PCV (%), postoperative PCV (%), requirement of a blood transfusion (including amount) were compared between the two groups (combined approach ± preligation) using a two-tailed student's *t*-test and boxplot graphs. Immediate postoperative complications (<48 h), short-term complications (48 h to 4 weeks), and clean histopathological margins were compared between the two groups (combined approach ± preligation) using a one-way ANOVA test. Pearson correlation analyses were performed between dog size and tumor size, in relation to duration of surgery and requirement of an intraoperative blood transfusion. All analyses were performed with commercially available software (GraphPad Prism8, San Diego, CA, USA), and values of *P* < 0.05 were considered statistically significant.

## Results

### Retrospective Case Series

Ninety-one maxillectomies were identified. Sixty-nine (76%) dogs had IO resections and these maxillectomies were not included in this study. Twenty-two (24%) animals had undergone caudal maxillectomy via a combined approach. Of these 22 dogs, six (27%) were performed via a traditional DL-IO procedure between 2004 and 2007 without preligation of the maxillary artery prior to osteotomies, and 16 (73%) were performed via a modified DL-IO approach between 2004 and 2018 with preligation of the maxillary artery prior to maxillectomy.

For the combined approach (*n* = 22), the most commonly reported breeds included the Labrador Retriever (*n* = 5), English Bulldog (*n* = 2), Golden Retriever (*n* = 2), Husky (*n* = 2) and German Shepherd (*n* = 2). Twelve dogs were female (one intact and 11 spayed) and 10 were male (one intact and nine neutered). Body weight and age ranged from 5.2 to 42.1 kg (median, 28.1 kg; mean, 26.5 ± 11.3 kg) and 1.5 to 14 years (median, 8.1 years; mean, 8 ± 3.5 years), respectively.

The right caudal maxilla (*n* = 19; 86%) was the most common location for the combined approach, followed by the left caudal maxilla (*n* = 3; 14%). These tumors often encompassed the premolar teeth and extended caudally to include the last molar tooth, as well as the rostral zygomatic arch and ventral orbit (*n* = 18; 81%). The total number of teeth removed ranged from three to nine (median, five teeth). Tumor types included acanthomatous ameloblastoma (*n* = 6; 27%), osteosarcoma (*n* = 4; 18%), fibrosarcoma (*n* = 4; 18%), and 1 (5%) each of the following tumor types: malignant melanoma, peripheral odontogenic fibroma, keratinizing ameloblastoma, multilobular tumor of bone, amyloid-producing odontogenic tumor, myxosarcoma, rhabdomyosarcoma, and squamous cell carcinoma. Based upon CT measurements, maximum tumor diameter ranged from 1.5 to 9 cm (median, 4 cm; mean, 4.2 ± 1.9 cm) by 1 to 7 cm (median, 4 cm; mean, 3.8 ± 1.4 cm) in the dorsoventral and mediolateral directions, respectively.

Six (27%) dogs had concurrent enucleation performed at the time of combined approach, and 16 (73%) did not. Of the six dogs that required enucleation, four had a traditional DL-IO approach without preligation of the maxillary artery and these were performed early in the study period between 2004 and 2006. The other two enucleations were performed in conjunction with the modified DL-IO approach between 2010 and 2018. The mean surgical time for the DL-IO and modified DL-IO approaches were 245 ± 69 and 196 ± 56 min, respectively. There was no significant difference in surgical times between the two types of procedures (*P* = 0.107), and there was no correlation in surgical duration when compared to dog size (*r* = 0.24, *P* = 0.287) or tumor size (*r* = 0.04, *P* = 0.856). Finally, there was no difference in surgical duration for cases that did (mean, 200.5 ± 55.3 min) and did not (mean, 212.1 ± 65.7 min) have an enucleation performed (*P* = 0.705).

In the traditional DL-IO group, four of the six (67%) animals required intraoperative blood transfusions. Two of these dogs required multiple units of intraoperative packed red blood cells (pRBCs), and one required an additional postoperative transfusion. Osteotomies were completed with a high-speed handpiece and rotary bur in five animals, and an oscillating bone saw in one animal. There was no association between bone cutting instrument and requirement for an intraoperative blood transfusion (*P* = 0.294). In this group, preoperative and postoperative PCV ranged from 40 to 54% (median, 43%; mean, 44.5 ± 5.1%) and 23 to 44% (median, 32.5%; mean, 32.7 ± 7.1%), respectively. The mean drop in PCV was 11.8 ± 9.8%. All six (100%) dogs in this group developed hypotension under anesthesia with a mean blood pressure <70 mmHg for >5 min.

**Figure 2:**
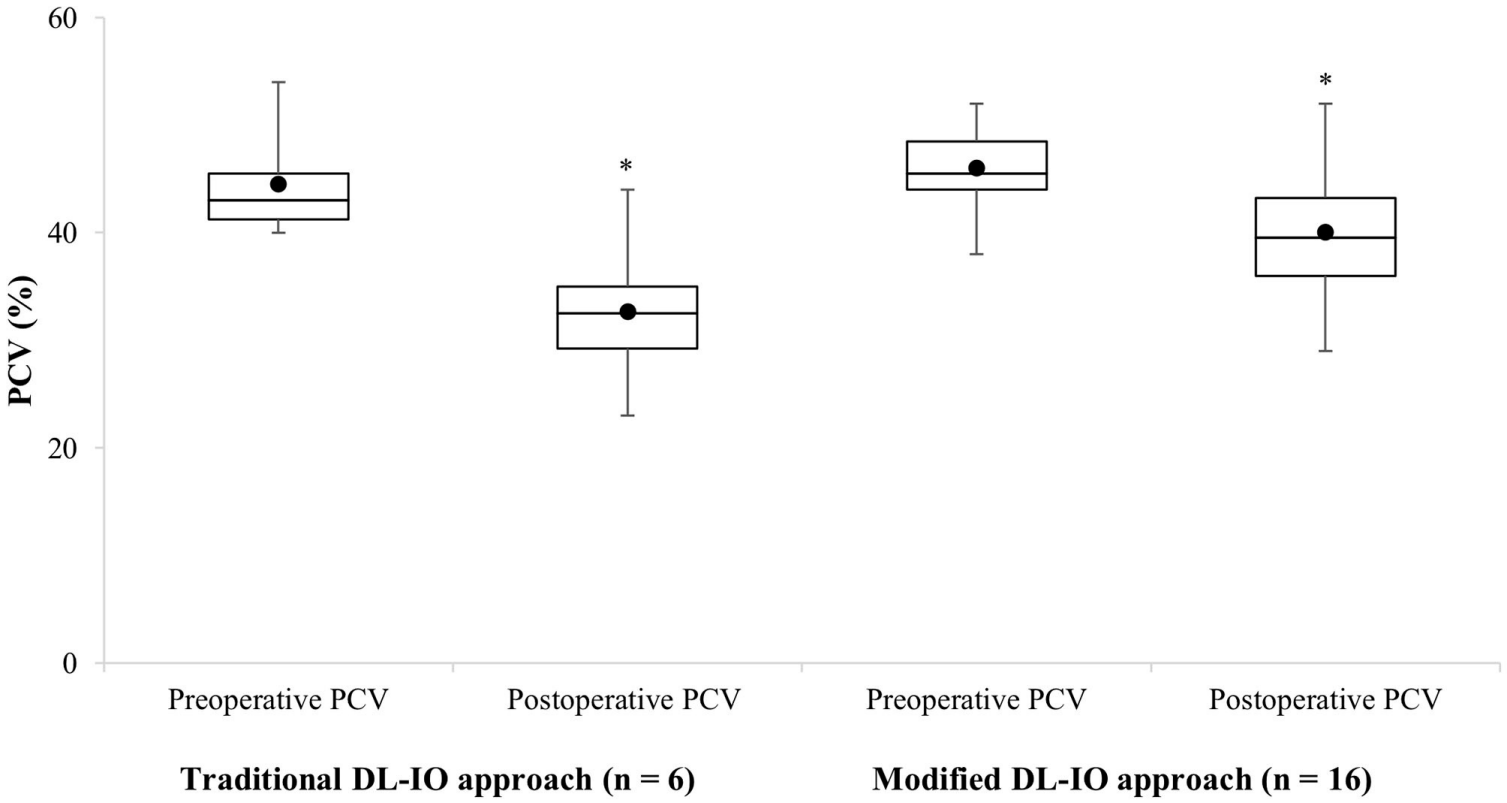
Boxplot representation of the preoperative and postoperative changes in PCV in 22 dogs undergoing caudal maxillary via a combined approach (traditional DL-IO approach, *n* = 6, four animals received intraoperative packed red blood cell transfusions; modified DL-IO approach with preligation of the maxillary artery, *n* = 16, one animal received an intraoperative packed red blood cell transfusion). The horizontal line denotes the median, the boxes represent cases within the 25–75th percentile, and the upper and lower bars represent the highest and lowest observed values that are not outliers. Asterisk (^*^) denotes significant difference.

In contrast, only one of the 16 (6%) dogs in the modified DL-IO group required an intraoperative blood transfusion. The preoperative PCV for this patient was 50%. Moderate hemorrhage was observed intraoperatively and the PCV dropped to 32%, necessitating the requirement of two units of pRBCs. Osteotomies were completed with a high-speed handpiece and rotary bur in 13 animals, and an oscillating bone saw in three animals. There was no association between bone cutting instrument and requirement for an intraoperative blood transfusion (*P* = 0.395). Of the 16 dogs in this group, preoperative and postoperative PCV ranged from 38 to 52% (median, 45.5%; mean, 46 ± 3.7%) and 39 to 48% (median, 39%; mean, 39.2 ± 4.9%), respectively. The mean drop in PCV was 6.8 ± 5.3%. Only three of 16 (19%) dogs in this group developed hypotension with a mean blood pressure <70 mmHg for >5 min. When compared, no significant differences were found between surgical approach (DL-IO vs. modified DL-IO) and preoperative PCV (*P* = 0.456) or drop in PCV (*P* = 0.135). However, a significant association was detected between postoperative PCV between the two groups (*P* = 0.021) with the postoperative PCV being higher in the maxillary artery preligation group, as compared to dog's treated with the traditional DL-IO approach (Figure 2). There was no correlation in terms of the requirement for an intraoperative blood transfusion when compared to dog size (*r* = 0.05, *P* = 0.838) or tumor size (*r* = 0.09, *P* = 0.700).

The most common immediate postoperative (<48 h) complications included facial swelling (*n* = 17; 11 with, and six without preligation), epistaxis (*n* = 14; eight with, and six without preligation), and facial pawing (*n* = 2; 0 with, and two without preligation). While facial swelling was most common (77%), this complication was self-limiting and resolved with supportive care. The most common short-term (48 h to 4 weeks) complications included oronasal fistula formation (*n* = 5; three with, and two without preligation), wound dehiscence (*n* = 4; two with, and two without preligation), lip ulceration (*n* = 3; one with, and two without preligation), and orbital swelling (*n* = 2; two with, and 0 without preligation). All five dogs with oronasal fistulas required revision surgery involving a buccal mucosal flap. Clean histopathological margins were obtained in 16 of 22 (73%) dogs as a result of the initial procedure. All six patients with incomplete surgical margins were treated with adjunct therapy including a combination of radiation and chemotherapy. When compared, no significant differences were found between surgical approach (DL-IO vs. modified DL-IO) and immediate postoperative complications (*P* = 0.224), short-term complications (*P* = 0.341), or clean histopathological margins (*P* = 0.124). Of the 22 dogs in the study, five had died by 6 months postoperatively. Each of these dogs had incomplete surgical margins and were euthanized for local disease recurrence (*n* = 2), pulmonary metastases (*n* = 2), or other medical conditions (*n* = 1; gastric dilatation and volvulus).

### Cadaveric Evaluation

In the 20 latex injected canine cadaver heads, three different spatial relationships of the arteries to the nerves were identified:

#### Version 1

All arteries remained immediately lateral to ventrolateral, to the nerves (maxillary, caudal superior alveolar, infraorbital and middle superior alveolar nerves) throughout their course (Figures 1A,B). Seven out of 20 sides (35%) (five left and two right) exhibited this neurovascular relationship. The infraorbital artery remained ventrolateral to the infraorbital nerve and its branches throughout the infraorbital canal.

#### Version 2

The arterio-neural spatial relationship was similar to version 1 except that the infraorbital artery rostral to the descending palatine artery, laid ventral and then medial to the ventral half of the infraorbital nerve as they entered the maxillary foramen. Within the infraorbital canal after the infraorbital nerve gave off the middle superior alveolar branches ventrally, the infraorbital artery laid ventrolateral to the infraorbital nerve. Additionally, the maxillary artery prior to giving off the minor palatine artery was just ventral to (contacting) the maxillary nerve, rather than lateral or ventrolateral to it (Figure 1C). Twelve out of 20 sides (60%) (four left and eight right) exhibited this neurovascular relationship.

#### Version 3

In one sample (5%) (left sided) the previously noted shift of the artery to a medial position occurred even earlier in its course (Figure 1D). The dorsal aspect of the maxillary artery laid medial to the maxillary nerve caudally. All arterial branches including the minor, descending, and major palatine, sphenopalatine and infraorbital arteries laid medial to the corresponding nerves. The infraorbital artery remained medial to the infraorbital nerve and its branches within the infraorbital canal.

In all specimens, the infraorbital and descending palatine arteries were of similar diameter and were the two largest branches of the maxillary artery.

## Discussion

Preligation of the maxillary artery resulted in less hemorrhage and subsequent need for blood transfusion when compared to historical data (4, 5, 9, 13) and when compared to caudal maxillectomy at this institution without preligation. Additionally, the spatial relationship of the maxillary artery and nerve branches was clarified and should aid surgeons performing this technique. Our overall findings support the safety and efficacy of this technique in caudal maxillectomy in dogs.

In six dogs, the maxillary artery was not preligated prior to caudal maxillectomy. Intraoperative hemorrhage was considered excessive in four of the six (67%) animals in this group who required intraoperative blood transfusions. Two of these dogs required multiple intraoperative transfusions, and one required an additional blood transfusion postoperatively. All six (100%) animals developed hypotension under anesthesia. These findings correspond with previous reports in the literature (4, 5, 9, 13). In contrast, results were more favorable for dogs that had preligation of their maxillary artery performed prior to caudal maxillectomy. Specifically, one out of 16 (6%) dogs required an intraoperative blood transfusion, and only three (19%) animals developed hypotension. A difference was identified between the two surgical approaches and postoperative PCV (*P* = 0.021; Figure 2). As four of the six dogs without preligation required an intraoperative transfusion, it is likely that the postoperative PCV difference between the two techniques would have actually been greater. These results suggest that preligation of the maxillary artery should be performed whenever possible, either through an enucleation prior to maxillectomy or via zygomatic ostectomy.

Zygomatic ostectomy allows access to the maxillary artery and nerve in the rostral aspect of the orbit. The rostral osteotomy can be performed close to the tumor to improve identification of these structures as long as the saw blade or bur used to make this cut is changed prior to other osteotomies to decrease the risk of tumor seeding. The zygomatic ostectomy is completed by making a second osteotomy caudal to the first and the section of the zygomatic bone is submitted for caudal margin histopathological analysis. Removal of this section of bone allows the surgeon improved exposure and facilitates retraction of the globe. Given the previous limited descriptions of the relationship of the maxillary artery to the nerve, the vascular clips were placed across all structures running horizontally into the maxillary foramen. All ligated structures including the maxillary nerve were transected at the end of the procedure, so having a clip that crosses both the artery and nerve was not an issue and we saw no complications postoperatively. The loose areolar connective tissue around the maxillary artery and nerve were easily separated in the cadaveric specimens using blunt dissection. Now that we have better defined the neurovascular anatomy of this area, it may be possible for surgeons to dissect the artery away from the nerve for more focused clip placement if desired. It is not known if preoperative assessment of the spatial relationship between the maxillary artery and nerve is possible, or which imaging modality would be most useful.

Regardless of maxillary artery preligation, the risk of hemorrhage during caudal maxillectomy may also vary depending on several other factors including surgeon experience (e.g., surgical resident, board-certified surgeon or dentist, oral and maxillofacial surgery fellow, years in practice, number caudal maxillectomies performed), preoperative diagnostic imaging and surgical planning, size of the tumor, size of the patient and breed, and availability and selection of surgical instrumentation. For this current study, all procedures were performed by a board-certified surgeon and a preoperative CT was performed prior to all caudal maxillectomies. Likewise, in similar studies where hemorrhage was also an important factor, a primary boarded surgeon and faculty member were consistently listed for each procedure (4, 5, 9, 13) and CT imaging was performed prior to all surgeries (4, 5, 9). However, it is acknowledged that the risk of hemorrhage may increase with less surgical training and experience, as well as lack of preoperative imaging. The majority of breeds in this study were mesocephalic, with the Labrador Retriever being most common (*n* = 3). Only three (14%) brachycephalic breeds were included in the study, and while head conformation may influence the risk of hemorrhage, the authors cannot comment further on this. Finally, it is possible that both dog size and tumor size may influence the risk of hemorrhage during caudal maxillectomy. However, in this study no statistically significant correlations in dog size or tumor size were detected in relation to surgical duration (*r* = 0.24, *P* = 0.287; *r* = 0.04, *P* = 0.856) or requirement for an intraoperative blood transfusion (*r* = 0.05, *P* = 0.838; *r* = 0.09, *P* = 0.700).

Two different types of bone cutting instruments were used in the present study including a high-speed handpiece and rotary bur (*n* = 18), and an oscillating bone saw (*n* = 4). Of the four caudal maxillectomies performed with an oscillating bone saw, two required an intraoperative blood transfusion. When further evaluated, there was no association between osteotomy instrument and the need for an intraoperative blood transfusion (traditional DL-IO group, *P* = 0.294; modified DL-IO group, *P* = 0.395). In terms of the veterinary literature, an oscillating saw is the predominant bone cutting device reported in caudal maxillectomy patients (4, 5, 9, 13). While the original DL-IO technique was described using an oscillating bone saw (9), it can be difficult to control fine movements with this instrument, and it has the potential to cause inadvertent and catastrophic damage to the neurovascular bundle (4, 15). The majority of caudal maxillectomies in the present study were performed using a high-speed handpiece and rotary bur. Irrigation was provided with sterile saline throughout the procedure, and these instruments allow for one cortex to be cut at a time and more precise osteotomies than an oscillating bone saw (4, 15). In human oral surgery, high speed handpieces are often contraindicated as they can generate inadvertent heat and can cause tissue emphysema and thermal necrosis (15). However, this complication has not been documented in animals, and was not seen in any of the dogs in the present study (16).

An alternative to these bone cutting devices is the piezoelectric oral surgical unit (17). Piezoelectric bone surgery is a recent and innovative technology in veterinary medicine where incisions are achieved through microvibrations (4). This instrument selectively cuts mineralized tissue at frequencies of 25–35 kHz, and prevents the user from inadvertently transecting nerves, blood vessels and soft tissue (17, 18). This thereby allows for precise osteotomies, excellent visibility within the surgical field, and significant reduction of soft-tissue trauma (17, 18). This technology was first developed for human dental and maxillofacial surgery (17), and its clinical application in people has since expanded to include neurosurgery, orthopedic surgery, as well as ear, nose and throat surgery (18, 19). However, piezoelectric bone surgery is less commonly documented in the veterinary literature (20–22), and while it would have been advantageous for cases in the present study, it is not currently available at the author's veterinary institution.

Knowledge of the spatial relationship of the maxillary artery and its branches to the maxillary nerve just caudal to the infraorbital canal may be important to surgeons performing caudal maxillectomy and to anyone performing maxillary nerve blocks prior to oral surgery. To our knowledge, this relationship has not been previously described in dogs, although it is illustrated in one canine text (10). This illustration, and a photograph from a single cadaver in another text (23) show the second most common relationship noted in the current study (Version 1; Figures 1A,B). Because cadaveric hemisections were used in this study, we were not able to evaluate potential asymmetry in anatomy between sides. Images from feline and equine anatomy articles and text books demonstrate the maxillary artery lateral to the maxillary nerve just caudal to the maxillary foramen, with no discussion regarding their spatial relationship or description of possible variations (24–26).

**Figure 3:**
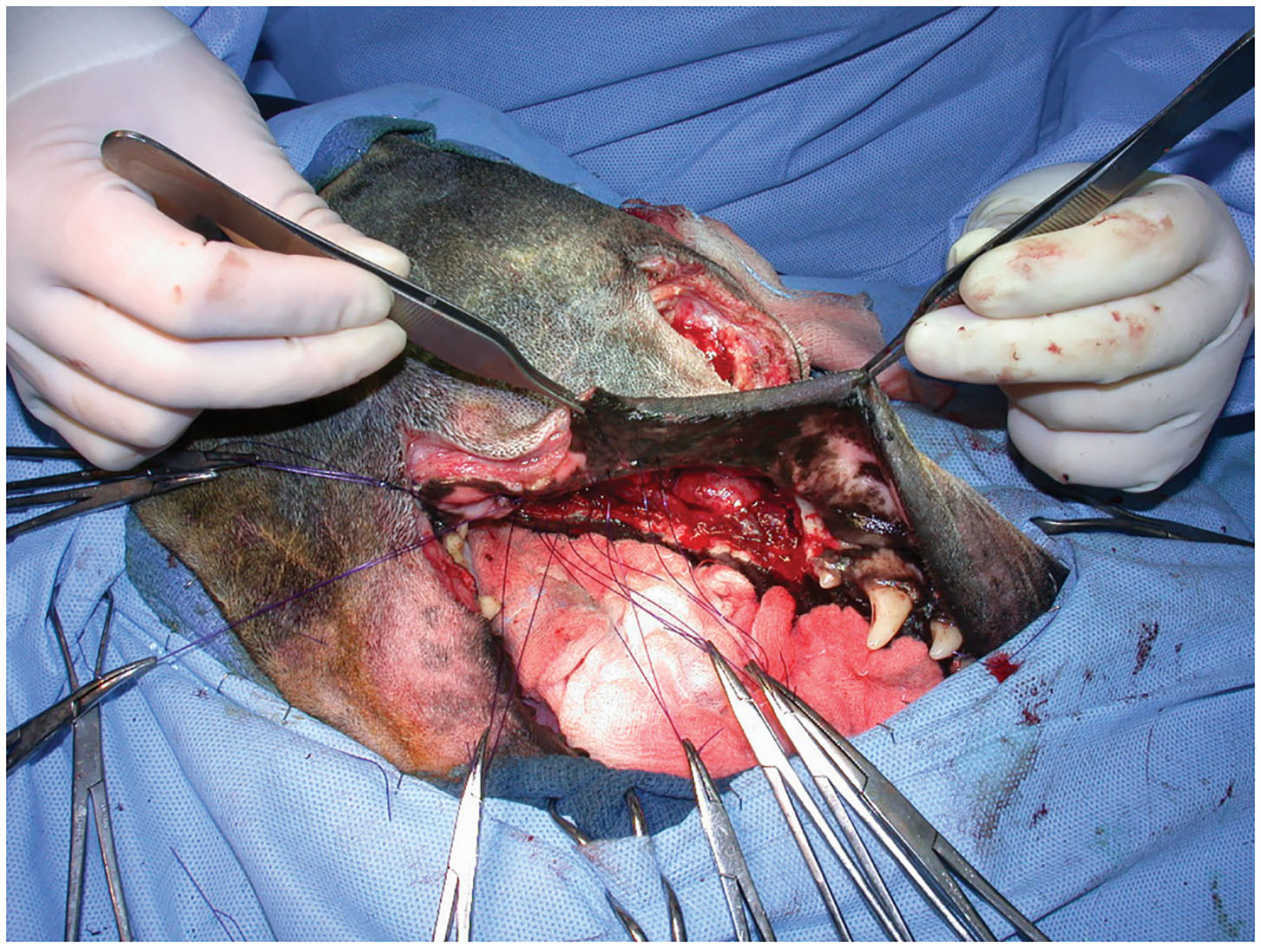
Intraoperative photograph following arterial preligation and caudal maxillectomy via a combined approach. Note the lack of hemorrhage on the surgical drape.

Relative diameters of the maxillary artery and its branches in this area are reported to be approximately 4 mm (maxillary artery), 0.5 mm (minor palatine artery), 1 mm (major palatine artery), and 2 mm (sphenopalatine artery), respectively in a medium sized dog (11). The infraorbital and descending palatine arteries are reportedly of similar diameter (11). Given the similar diameter of the infraorbital and descending palatine arteries, diminution of blood flow to the maxilla would be greatest if both branches were occluded, or if the maxillary artery was occluded caudal to their origins. The location of hemoclip application was not recorded for the retrospective cases reported. Regardless, blood loss was found to be diminished compared to previous reports given that only three dogs with maxillary artery preligation developed intraoperative hypotension and one dog required an intraoperative blood transfusion (Figure 3). This would appear to be an improvement over the 30–50% of dogs requiring intraoperative transfusions as reported in the literature (4, 5, 9, 13). In the one dog that required an intraoperative blood transfusion in the modified DL-IO group, a large degree of hemorrhage was reported following osteotomy with an oscillating bone saw. After discussing the case with the surgeon, they felt that the vascular clips may not have been fully across the maxillary artery or its branches, as additional clips were necessary at that location following tumor resection. Therefore, this hemorrhage was likely the result of inadequate hemoclip placement across the maxillary artery.

Preemptive arterial ligation to decrease the risk of intraoperative hemorrhage in maxillofacial surgery has a precedent in the veterinary literature, including ligation of the carotid artery when local measures or direct ligation of the bleeding vessel is not possible. Unilateral or bilateral common carotid artery ligations have been demonstrated to decrease lingual arterial pressure in the dog (14). However, no studies have evaluated the effectiveness of carotid artery ligation in reducing hemorrhage during maxillectomies. Carotid ligation requires a separate surgical approach to the cervical region and can be performed with no notable adverse effects due to the well-developed collateral circulation system in the dog originating from the vertebral arteries (27–32). There are at least five described sites of anastomoses between the internal and external carotid arteries in the dog, allowing them to survive with no residual effect following long-term ligation of one or both carotid arteries and other major vessels supplying the head (27–32). Likewise, multiple studies have demonstrated that ligation of the maxillary, infraorbital and/or major palantine vessels to control intraoperative hemorrhage does not result in adverse effects (6, 13). Circulation to the face and oral tissues is maintained by facial artery branches and contralateral maxillary and ethmoidal vasculature (9, 11). It is not known if maxillary artery ligation would eventually compromise structures especially the teeth, rostral to the surgical site. Although this retrospective study focused on intraoperative and short-term complications (and lacked long-term follow-up >1 year from the time of surgery), this complication was not identified in any of the animals during the study period.

As a retrospective study at a single academic referral hospital, several limitations are associated with the study presented here. The study was constrained by a limited sample size. Of the 22 dogs that met the inclusion data, only six and 16 were treated via a DL-IO and modified DL-IO approach, respectively. This variation in case numbers between the two procedures was likely due to different surgeon preferences over the long study period. Surgeon experience and overall comfort levels may have also affected decision making in terms of surgical approach. Enucleations described herein tended to be earlier in the study, reflecting the shift from two-dimensional and three-dimensional conformal radiotherapy to modern intensity-modulated radiotherapy, which is capable of super ocular-sparing (33). Concerns over including the eye in a potential radiation field likely had an influence on whether or not enucleation was performed.

In conclusion, this study demonstrates the effectiveness of preligation of the maxillary artery in limiting hemorrhage and the subsequent need for blood transfusions during caudal maxillectomy procedures in dogs. To the authors' knowledge, this is the first study to describe a modification of the existing DL-IO combined approach, and to clarify the regional vascular anatomy of the maxillary artery and nerve. Further prospective studies are indicated to evaluate the feasibility and safety of this approach in a larger cohort of clinical animals.

## Data Availability Statement

The original contributions generated for this study are included in the article/Supplementary Material, further inquiries can be directed to the corresponding author/s.

## Ethics Statement

Ethical review and approval was not required for the animal study because, the data was collected retrospectively from medical records. Written informed consent for participation was not obtained from the owners because, there was no change in procedure other than the timing of arterial ligation.

## Author Contributions

KC: data collection, data analysis, manuscript composition, and final approval of the version to be published. KM: concept generation and design, data collection, data analysis, manuscript composition, and final approval of the version to be published. All authors contributed to the article and approved the submitted version.

## Conflict of Interest

The authors declare that the research was conducted in the absence of any commercial or financial relationships that could be construed as a potential conflict of interest.
