# Supplementary material for: Ligation of the Maxillary Artery Prior to Caudal Maxillectomy in the Dog—A Description of the Technique, Retrospective Evaluation of Blood Loss, and Cadaveric Evaluation of Maxillary Artery Anatomy
Source: Front Vet Sci. 2020 Nov 5;7:588945. doi: 10.3389/fvets.2020.588945 (PMC7674398; doi:10.3389/fvets.2020.588945)
Supplement: Supplementary file 1 [file Table_1.DOCX]

| **Case** | **Signalment** | **Weight (kg)** | **Clinical Signs and Duration** | **Tumor Type** | **Tumor Size (cm)** | **Tumor Location** | **No. of Teeth Removed (n)** | **Preligation of the Maxillary Artery?** | **Orbitectomy Performed?** | **Enucleation Performed?** | **Duration of Surgery (mins)** |
| --- | --- | --- | --- | --- | --- | --- | --- | --- | --- | --- | --- |
| 1 | 7-yr-old, FS Labrador Retriever | 29.4 | Swelling below right eye  4 months | Osteosarcoma | 5 x 3.5 | Right caudal maxilla | 7 | No | Yes | Yes | 245 |
| 2 | 7-yr-old, FS Husky | 28.4 | Swelling below right eye  1 month | Osteosarcoma | 3.2 x 3.2 | Right caudal maxilla | 4 | No | No | Yes | 215 |
| 3 | 1.5 yr-old, MI English Bulldog | 27.8 | Facial rubbing  5 months | Peripheral odontogenic fibroma | 5 x 5 | Right caudal maxilla | 7 | No | No | No | 370 |
| 4 | 1.5 yr-old, FI English Bulldog | 21.6 | Swelling below left eye 3 weeks | Osteosarcoma | 5 x 5 | Left caudal maxilla | 4 | No | Yes | Yes | 260 |
| 5 | 8 yr-old, FS Labrador Retriever | 32.4 | Swelling of right eye  1 month | Acanthomatous ameloblastoma | 2 x 4 | Right caudal maxilla | 4 | No | No | No | 175 |
| 6 | 10 yr-old, MN Labrador Retriever | 16 | Swelling of right eye  1 month | Fibrosarcoma | 9 x 4 | Right caudal maxilla | 7 | No | No | Yes | 198 |
| 7 | 11 yr-old, FS Australian Shepherd | 23.2 | Swelling below right eye  1 month | Acanthomatous ameloblastoma | 4 x 3 | Right caudal maxilla | 5 | Yes | No | No | 260 |
| 8 | 8 yr-old, MN Boston Terrier | 8 | Swelling below right eye  3 months | Acanthomatous ameloblastoma | 5 x 4 | Right caudal maxilla | 7 | Yes | No | No | 210 |
| 9 | 7-yr-old, MN Border Collie | 41.5 | Swelling below right eye  1 month | Keratinizing ameloblastoma | 4 x 4 | Right caudal maxilla | 6 | Yes | No | No | 275 |
| 10 | 8-yr-old, MN Hound dog | 33.1 | Bleeding from mouth  1 month | Multilobular tumor of bone | 4 x 3.5 | Right caudal maxilla | 9 | Yes | Yes | Yes | 105 |
| 11 | 11-yr-old, FS mixed breed dog | 11.6 | Mass noted during routine examination  1 month | Acanthomatous ameloblastoma | 1.5 x 1 | Right caudal maxilla | 5 | Yes | No | No | 182 |
| 12 | 13-yr-old, FS Miniature Pinscher | 5.2 | Swelling below left eye 1 month | Amyloid-producing odontogenic tumor | 1.5 x 2 | Right caudal maxilla | 4 | Yes | Yes | No | 100 |
| 13 | 5-yr-old, MN mixed breed dog | 41 | Swelling below left eye 5 months | Myxosarcoma | 5 x 4.4 | Right caudal maxilla | 8 | Yes | Yes | No | 205 |
| 14 | 10-yr-old, MN German Shepherd | 42.1 | Swelling below right eye  1 month | Malignant melanoma | 6 x 5 | Right caudal maxilla | 6 | Yes | Yes | Yes | 180 |
| 15 | 1.25-yr-old, FS Labrador Retriever | 27.8 | Swelling below left eye 1 month | Rhabdomyosarcoma | 3 x 2.9 | Right caudal maxilla | 4 | Yes | No | No | 192 |
| 16 | 9-yr-old, MN Husky | 30.1 | Swelling below left eye 1 week | Acanthomatous ameloblastoma | 3 x 4 | Right caudal maxilla | 3 | Yes | Yes | No | 210 |
| 17 | 11-yr-old, FS Beagle | 15.6 | Swelling below right eye  3 months | Osteosarcoma | 3 x 3 | Right caudal maxilla | 5 | Yes | No | No | 151 |
| 18 | 8-yr-old, MN Golden Retriever | 38.1 | Swelling below right eye  2 months | Fibrosarcoma | 4 x 5 | Right caudal maxilla | 6 | Yes | No | No | 210 |
| 19 | 12-yr-old, MN German Shepherd | 35.5 | Swelling below right eye, difficulty eating  3 months | Fibrosarcoma | 4 x 6 | Right caudal maxilla | 4 | Yes | Yes | No | 267 |
| 20 | 8-yr, old, FS Labrador Retriever | 26 | Mass noted during routine examination  1 month | Squamous cell carcinoma | 3 x 1 | Left caudal maxilla | 5 | Yes | No | No | 280 |
| 21 | 5-yr-old, MN Golden Retriever | 38.4 | Swelling below right eye, difficulty eating  3 months | Fibrosarcoma | 9 x 7 | Right caudal maxilla | 5 | Yes | No | No | 147 |
| 22 | 14-yr-old, FS Pembroke Welsh Corgi | 10.1 | Swelling below left eye 1 month | Acanthomatous ameloblastoma | 4 x 3 | Left caudal maxilla | 4 | Yes | No | No | 160 |

**Table 1.** Signalment, history and surgical procedure performed on 22 dogs undergoing caudal maxillary via a combined approach. Cases 1-6 were performed via a traditional DL-IO approach to caudal maxillectomy. Cases 7-22 were performed via a modified DL-IO approach to caudal maxillectomy involving preligation of the maxillary artery.
