# Supplementary material for: Ligation of the Maxillary Artery Prior to Caudal Maxillectomy in the Dog—A Description of the Technique, Retrospective Evaluation of Blood Loss, and Cadaveric Evaluation of Maxillary Artery Anatomy
Source: Front Vet Sci. 2020 Nov 5;7:588945. doi: 10.3389/fvets.2020.588945 (PMC7674398; doi:10.3389/fvets.2020.588945)
Supplement: Supplementary file 2 [file Table_2.DOCX]

| **Case** | **Preligation of the Maxillary Artery?** | **Preoperative PCV (%)** | **Intraoperative PCV (%)** | **1-hour Postoperative PCV (%)** | **Intraoperative Blood Transfusion; Amount** | **Intraoperative Hypotension (Mean Blood Pressure < 70 for > 5 mins)?** | **Immediate Postoperative Complications (<48 hours)** | **Short-Term Complications (48 hours to 4 weeks postoperatively)** | **Resection Margins / Adjunct Therapy** |  |
| --- | --- | --- | --- | --- | --- | --- | --- | --- | --- | --- |
| 1 | No | 40 | 14 | 35 | Yes; 2 units | Yes | Facial swelling, facial pawing, epistaxis | Oronasal fistula, wound dehiscence | Clean / No |  |
| 2 | No | 54 | N/A | 30 | No | Yes | Epistaxis, facial swelling | Lip ulceration | Clean / Chemotherapy |  |
| 3 | No | 46 | N/A | 23 | Yes; 2 units | Yes | Epistaxis, facial swelling | Lip ulceration | Clean / No |  |
| 4 | No | 41 | 28 | 29 | Yes; 1 unit | Yes | Epistaxis, facial swelling, facial pawing | None | Clean / No |  |
| 5 | No | 44 | 28 | 44 | Yes; 1 unit | Yes | Epistaxis, facial swelling | None | Clean / No |  |
| 6 | No | 42 | N/A | 35 | No | Yes | Epistaxis, facial swelling | Oronasal fistula formation, wound dehiscence | Dirty / Radiation therapy |  |
| 7 | Yes | 44 | N/A | 44 | No | Yes | Epistaxis, facial swelling | None | Clean / No |  |
| 8 | Yes | 44 | N/A | 40 | No | No | Epistaxis, facial swelling | None | Clean / No |  |
| 9 | Yes | 52 | N/A | 46 | No | No | Epistaxis, facial swelling | None | Clean / Radiation therapy |  |
| 10 | Yes | 40 | N/A | 39 | No | No | Epistaxis, facial swelling | None | Clean / No |  |
| 11 | Yes | 48 | N/A | 29 | No | Yes | Epistaxis, facial swelling | Oronasal fistula formation, dehiscence | Clean / No |  |
| 12 | Yes | 45 | N/A | 35 | No | No | Epistaxis, facial swelling | Oronasal fistula formation, dehiscence | Clean / No |  |
| 13 | Yes | 45 | N/A | 36 | No | No | Epistaxis, facial swelling | None | Clean / Radiation therapy |  |
| 14 | Yes | 47 | N/A | 35 | No | No | None | None | Dirty / Radiation therapy |  |
| 15 | Yes | 48 | N/A | 43 | No | No | None | None | Dirty / No |  |
| 16 | Yes | 38 | N/A | 36 | No | No | Facial swelling | Left orbit swelling | Clean / No |  |
| 17 | Yes | 50 | N/A | 47 | No | No | Facial swelling | Right orbit swelling | Dirty / Chemo- and radiation therapy |  |
| 18 | Yes | 45 | N/A | 39 | No | No | None | None | Dirty / Chemotherapy |  |
| 19 | Yes | 50 | N/A | 36 | No | No | None | None | Dirty / Radiation therapy |  |
| 20 | Yes | 50 | N/A | 52 | Yes; 2 units | Yes | Facial swelling | Oronasal fistula | Clean / No |  |
| 21 | Yes | 46 | N/A | 41 | No | No | Facial swelling | Lip ulceration | Clean / Radiation therapy |  |
| 22 | Yes | 44 | N/A | 41 | No | No | Epistaxis | None | Clean / No |  |

**Table 2.** Intra-, and postoperative data on 22 dogs undergoing caudal maxillary via a combined approach. Cases 1-6 were performed via a traditional DL-IO approach to caudal maxillectomy. Cases 7-22 were performed via a modified DL-IO approach to caudal maxillectomy involving preligation of the maxillary artery. N/A denotes that information was unavailable.
